# Supplementary material for: Patchwork of contrasting medication cultures across the USA
Source: Nat Commun. 2018 Oct 9;9:4022. doi: 10.1038/s41467-018-06205-1 (PMC6177425; doi:10.1038/s41467-018-06205-1)
Supplement: Supplementary file 1 — Supplementary Information [file 41467_2018_6205_MOESM1_ESM.pdf]

Supplementary information

Patchwork of contrasting medication cultures across the USA

Melamed, et al.

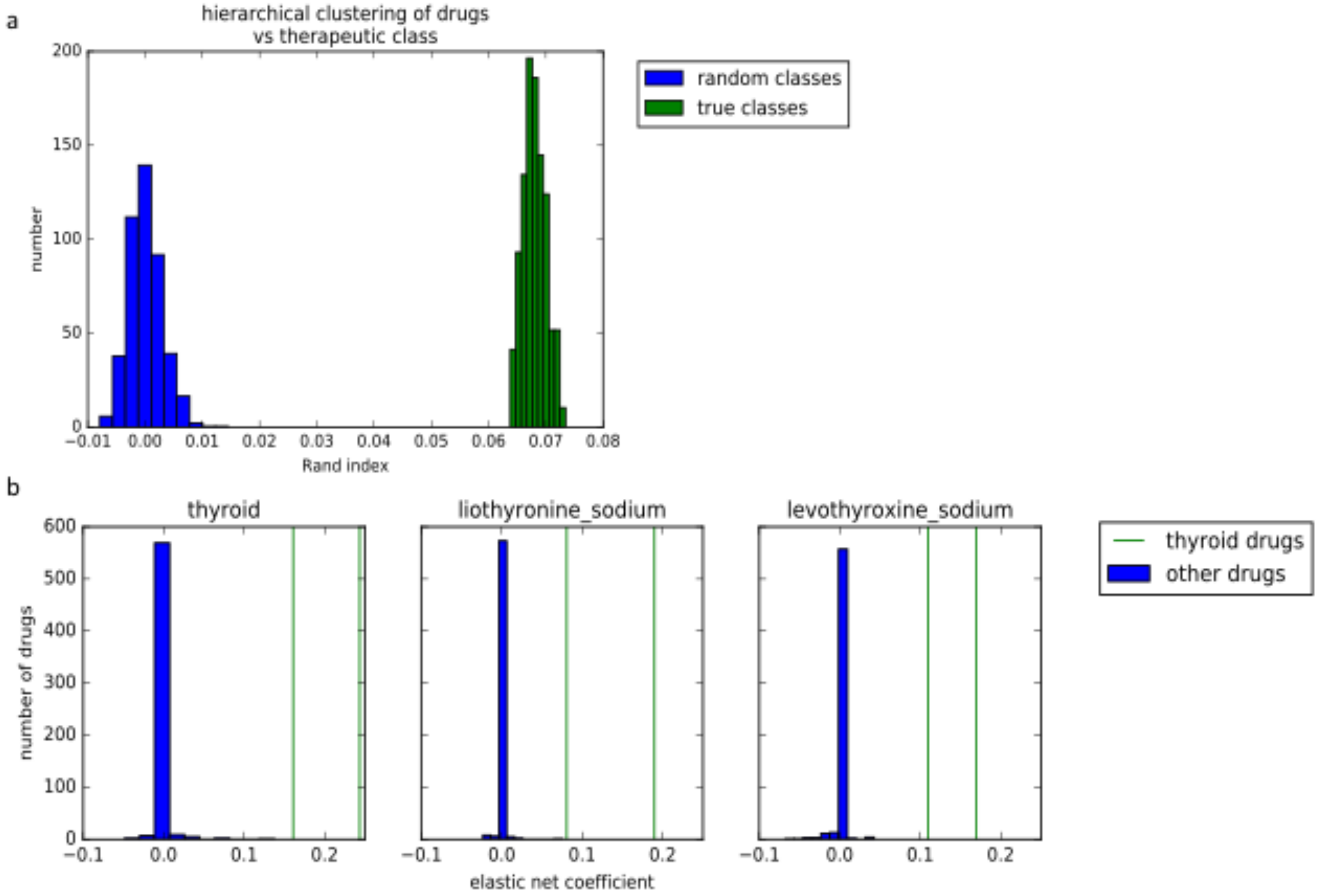

Supplementary Figure 1. Drugs in the same therapeutic class have similar county deviance vectors **a.** Rand index is used to compare drug therapeutic class against hierarchical clustering of drug deviance vectors. To assess the significance of the Rand index, we compare it against randomly permuted assignments of drug therapeutic classes. **b.** We predict deviance values each of the three popular thyroid hormone replacement drugs, using all other drugs as a predictors, in an elastic net model. For each, the elastic net model shrinks most coefficients to zero (blue histogram), and the other two thyroid drugs are the most predictive of the third drug.

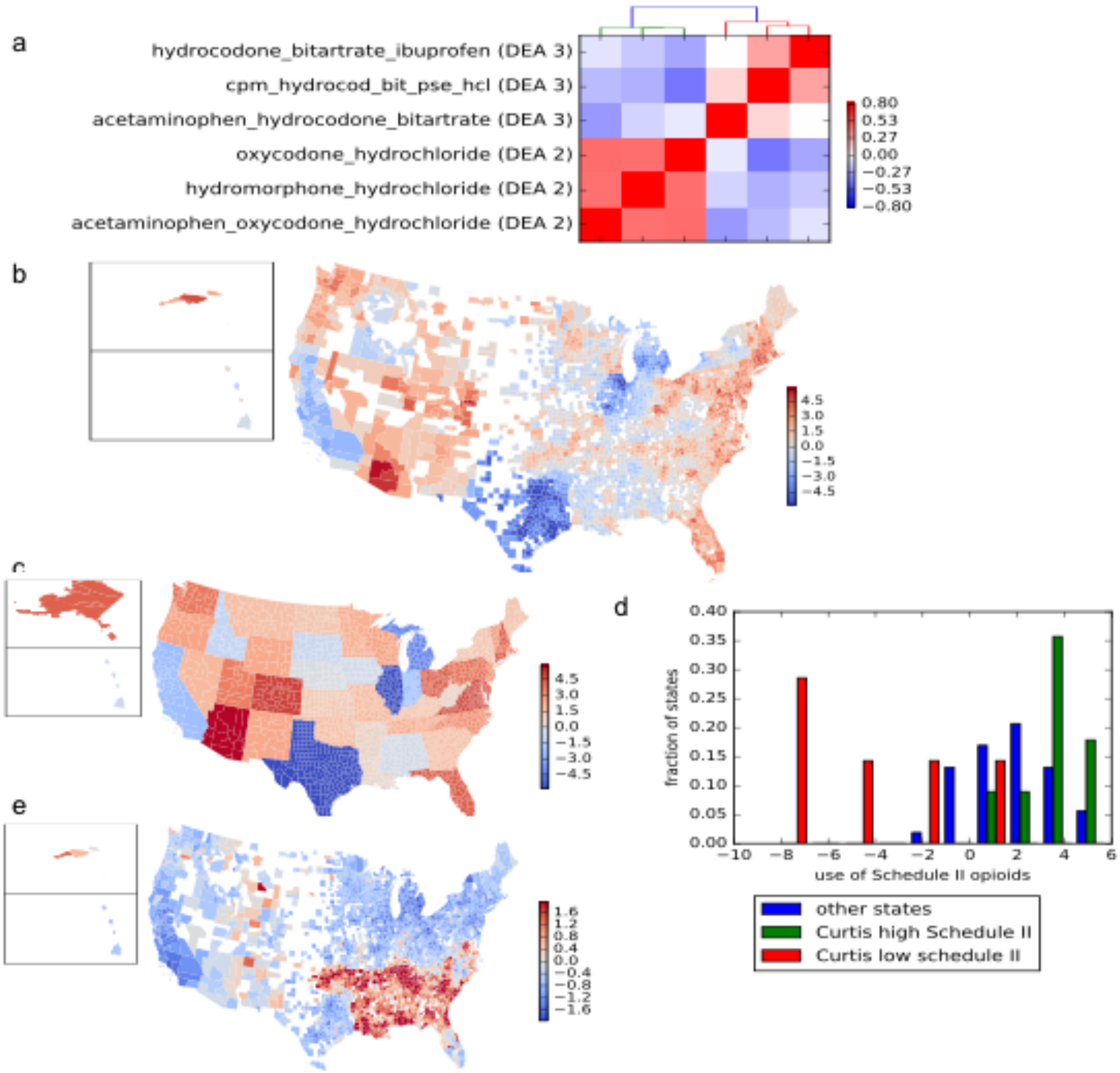

Supplementary Figure 2. Fig 2: Variation in schedule II and III opioid use **A.** Symmetric clustering of correlation between use of common DEA schedule II and III drugs **B.** Per-county mean drug deviance in use of the Schedule II opioids, where the mean of the opioids is weighted by number of prescriptions of each drug. **C.** State-level use of Schedule II opioids is shown, for comparison with Curtis 2006. **D.** Comparing deviance values for the states that Curtis 2006 identified as highest and lowest use of Schedule II opioids. **E.** Averaged deviance values for the highly correlated schedule II opioids meperidine_hcl_promethazine_hcl and meperidine_hydrochloride.

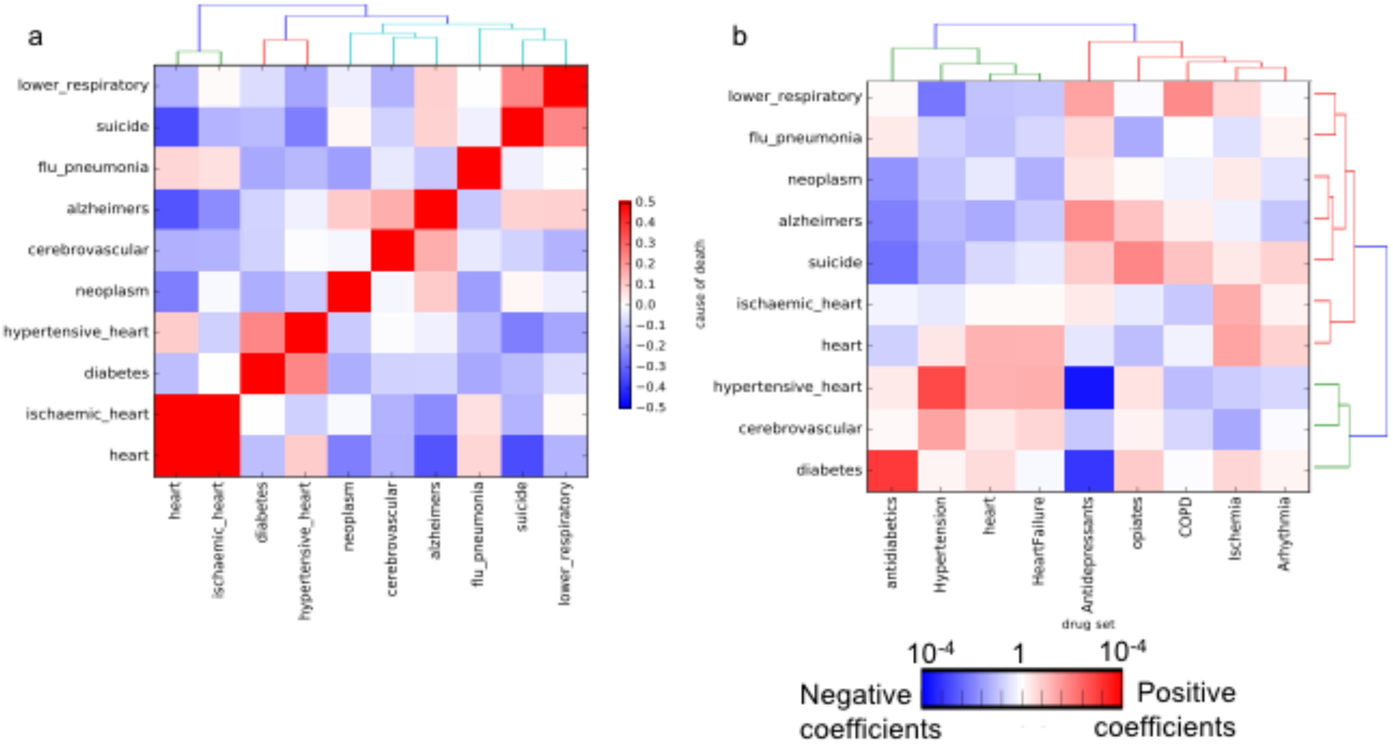

Supplementary Figure 3: Comparison of death rates with drug use. **a.** We cluster correlations across counties of rates of each cause of death, as a fraction of total age-adjusted death rate, reported from CDC (legend shows Spearman correlation). **b.** We predict death rates from drug deviances using regularized regression, and then evaluate whether drugs used to treat the condition have elevated coefficients using the two-sided rank sum test. The darkness of each box corresponds to the p-value of the test for each combination of death and drug set. Red boxes have elevated positive coefficients (the right tail of the test), while blue boxes have negative coefficients, indicating that the drug set is anticorrelated with the death cause. For each cause of death (row), the expected drug set, if any, has the highest positive set of coefficients.

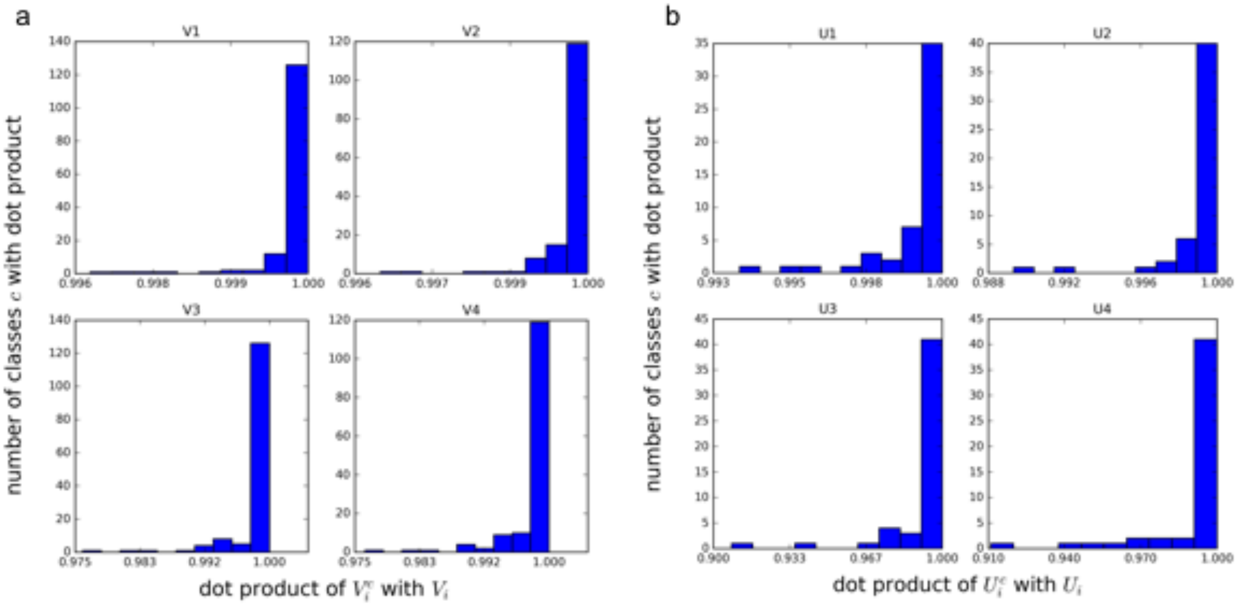

Supp Fig 4: Stability of the PCA. **a.** We remove each of 153 classes of drugs in turn, and calculate PCA. Then, we calculate the dot product of the PCA eigenvectors from the full PCA versus the PCA with down-sampled data, for each of the first four components. The distribution in each plot is across classes of drugs removed. **b.** Same, but removing one state at a time, and comparing the drug eigenvectors.

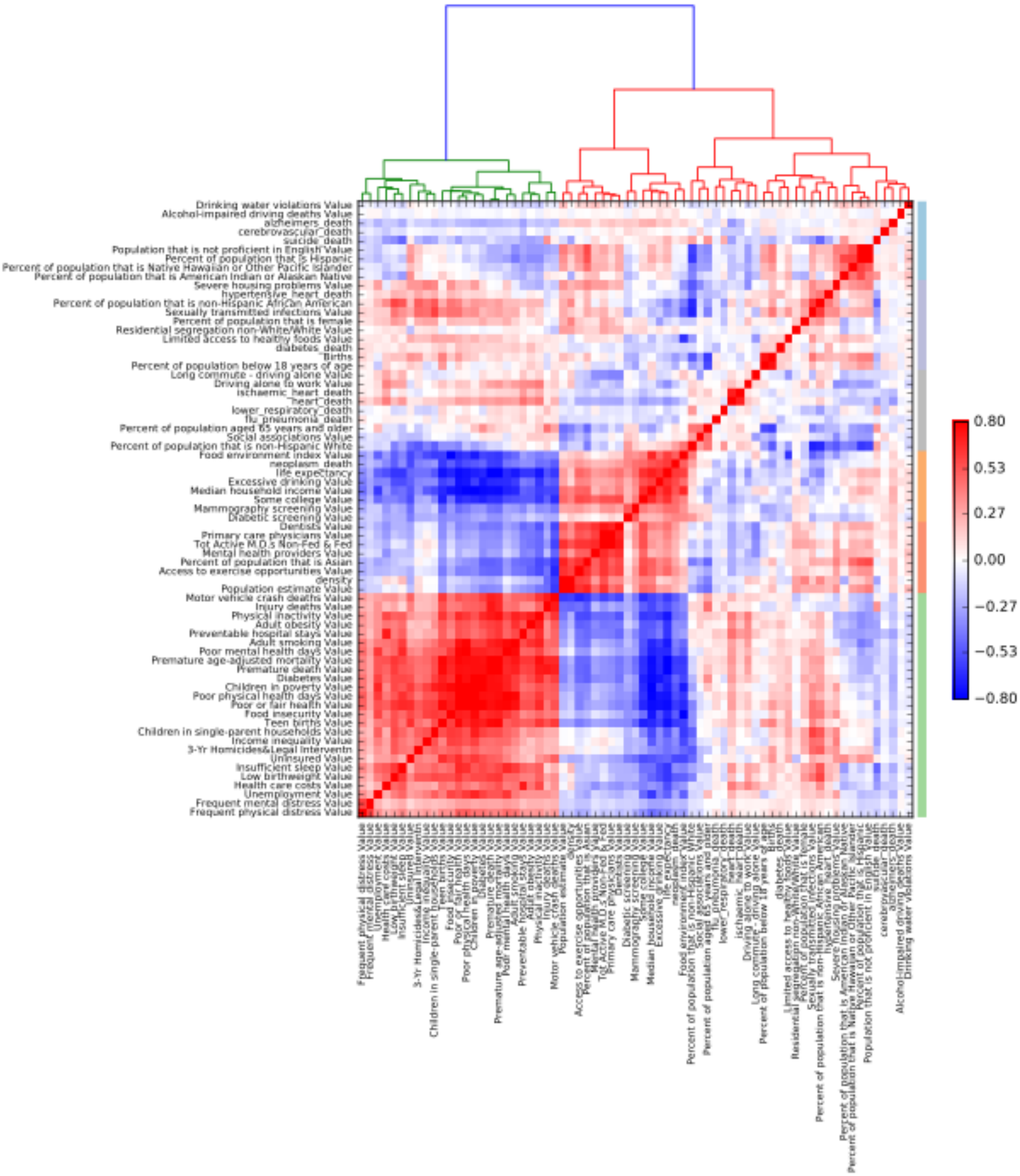

Supplementary Figure 5: Demographic indicators were compiled from sources described in Methods. We cluster them by their correlations across counties. The red-blue scale indicates spearman correlation (see colorbar). The other color bar corresponds to clusters identified by cutting the dendrogram. These colors, indicating correlated demographic variables, are used in Figure 3.

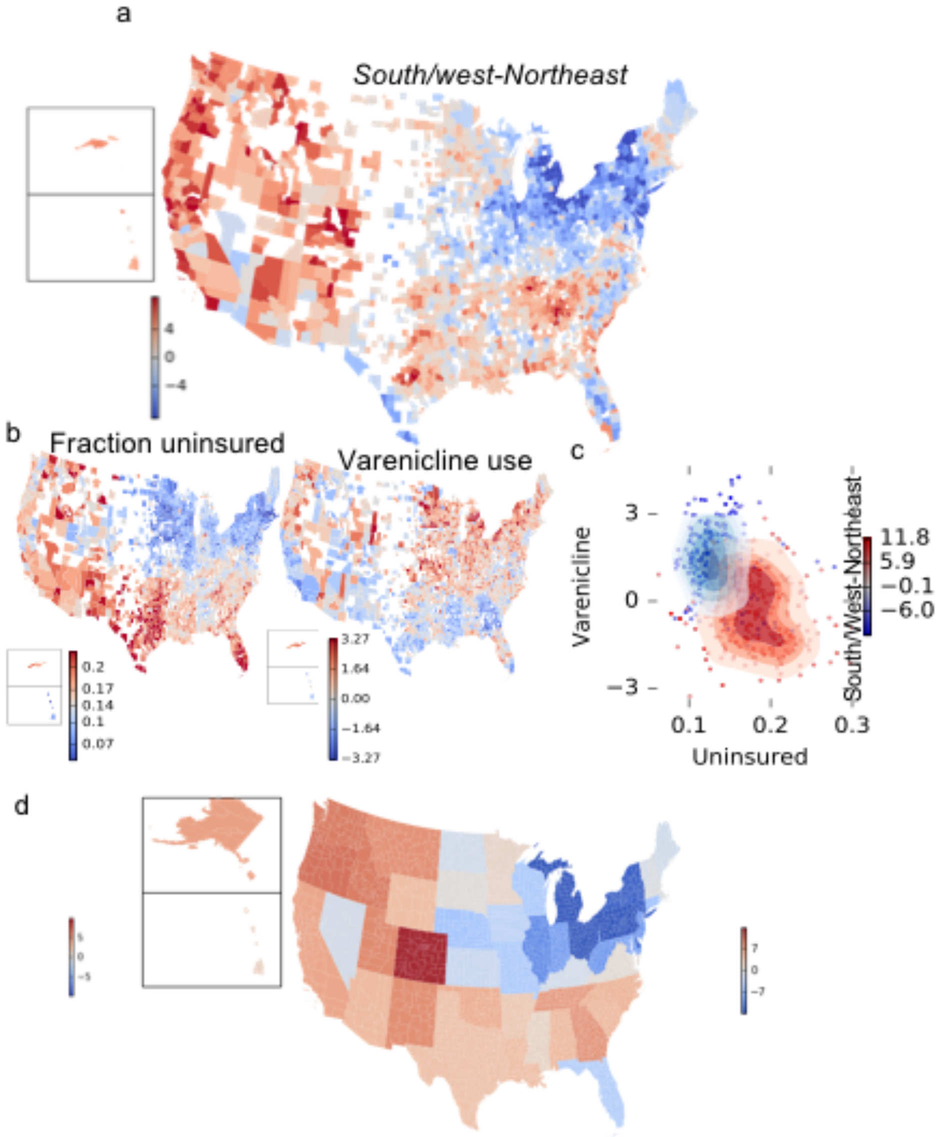

Supplementary Figure 6: Associations for *South/west-Northeast* **a.** Projected values for each county. **b.** *South/west-Northeast* is correlated with fraction uninsured (Spearman correlation = .36), among other demographic variables. Many of the drugs associated with this component have deviance values correlated with level of insurance, for example Varenicline. **c.** Comparison of counties at the extremes of *South/west-Northeast* in terms of Uninsured rates and varenicline deviance, with estimated density contours showing that the *Northeastern* counties have more insured and more varenicline. **d.** State projections for this component.

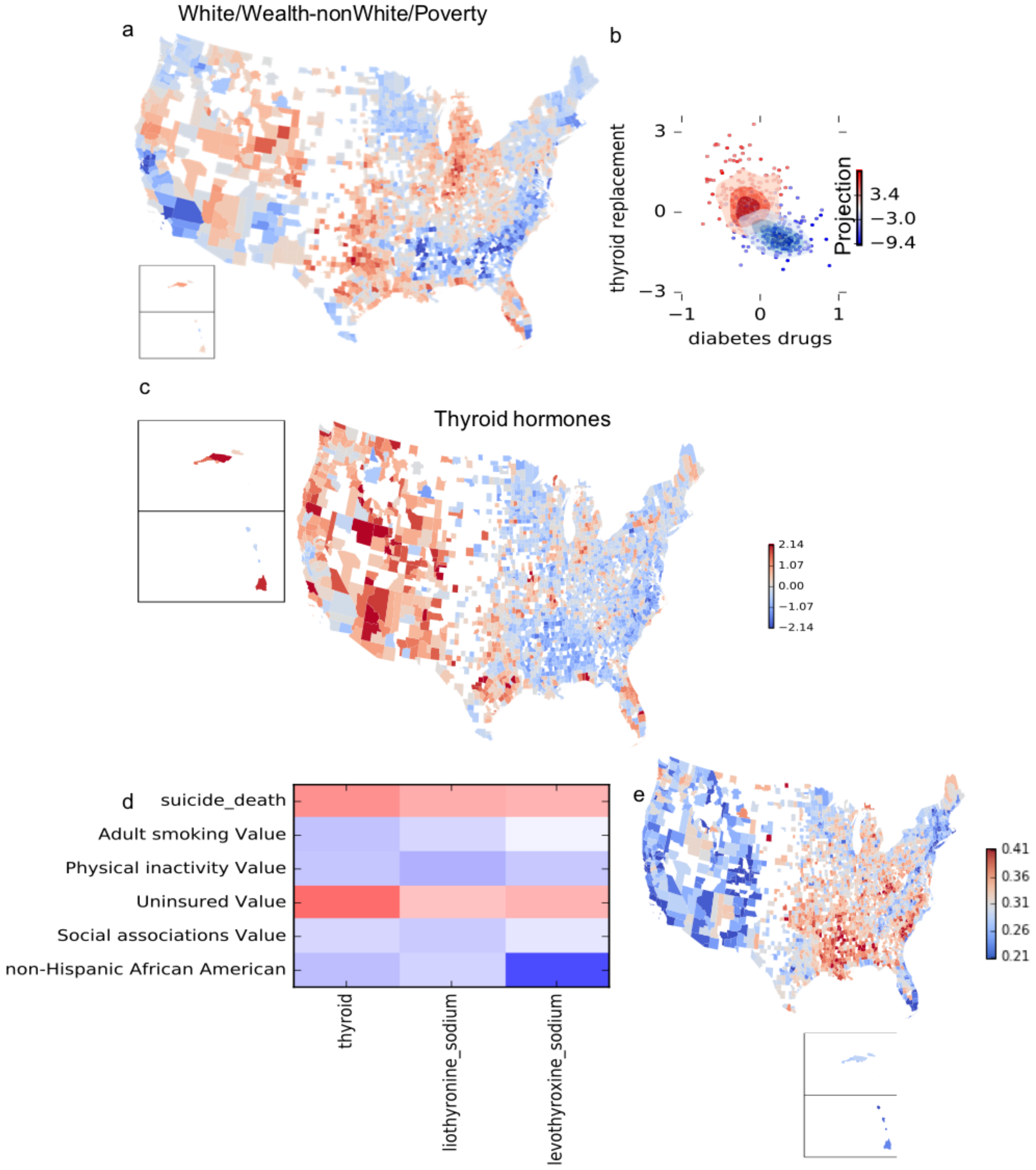

Supplementary Figure 7: Associations *for White/Wealth-nonWhite/Poverty* **a.** Projected values for each county. **b.** Use of diabetes drugs and thyroid hormone replacement for counties at the two extremes of this component. **c.** Deviance values for thyroid hormones, which are distinctly lower in the *nonWhite/Poverty* counties, and the eastern US in general. **d.** Coefficients for demographic variables used to predict the three thyroid hormones, fitted with a multi-task elastic net regression. **e.** Obesity per county (demographic data described in Methods).

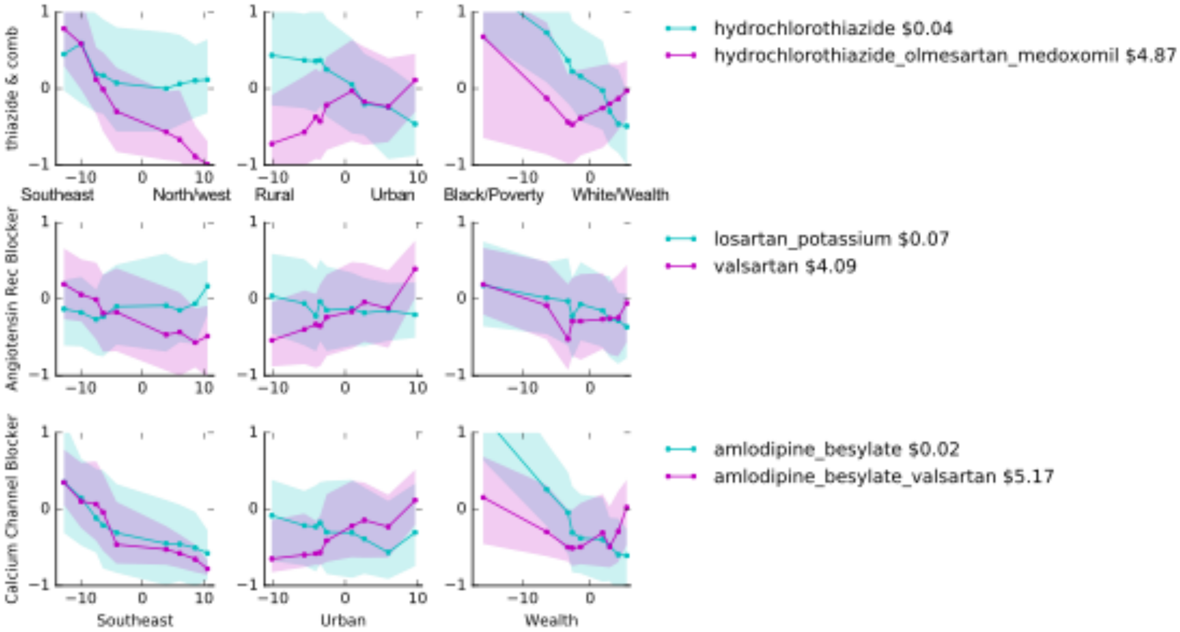

Supplementary Figure 8: Using the same scheme as in Figure 5F, we compare the three cost-associated components (columns), and three classes of antihypertension drugs with high cost variance. For each therapeutic class, we compare an expensive and a cheap drug. Again, the 25th to 75th percentiles of prescription in each bin of counties, grouped by projected value, are shown.

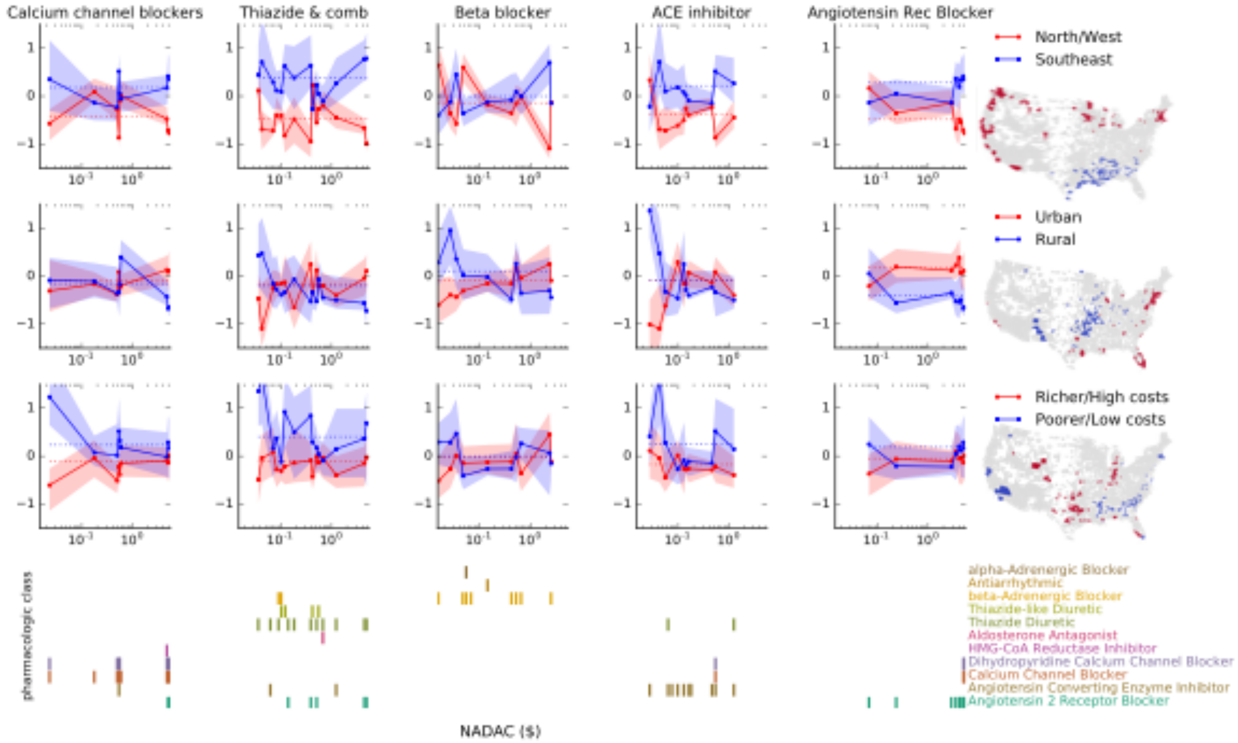

Supplementary Figure 9: Similar to Figure 5d,e, we compare use of hypertension drugs in the most extreme counties for each component against drug prices. We examine 5 drug classes. Instead of showing fraction brand, as in Figure 5, we show drug price (NADAC, in dollars). For example, the right-most columns, Angiotensin Receptor Blockers, shows that the Southeastern counties and urban counties prefer the more expensive drugs. The poor/low cost counties use less of these drugs, especially compared to their use of other hypertension drugs such as thiazides. The bottom panel shows the pharmacologic classes of the drugs in a column. For example, in the Thiazides & comb column, more expensive thiazides are mostly combination drugs combined with Angiotensin Receptor Blockers

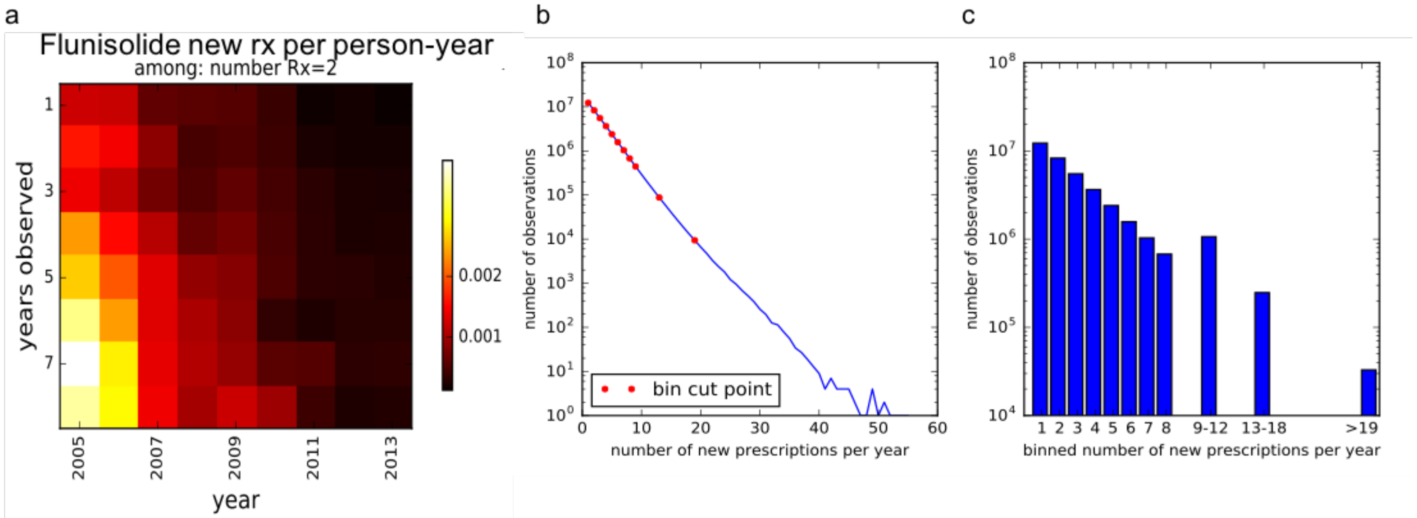

Supplementary Figure 10: Using rate of new prescriptions to predict prescription of a drug. **a.** The rate of prescription for an example drug, flunisolide, is shown among person-years with 2 new prescriptions. People with more years of prescription data have a higher rate of this drug, showing that this variable provides more information. **b.** The distribution of number of new prescriptions per year in our set of person-years. The points that we selected for binning the person-years are shown in red. We only bin person-years containing 9 or more new prescriptions. **c.** The total number of person-years falling into each of the bins.

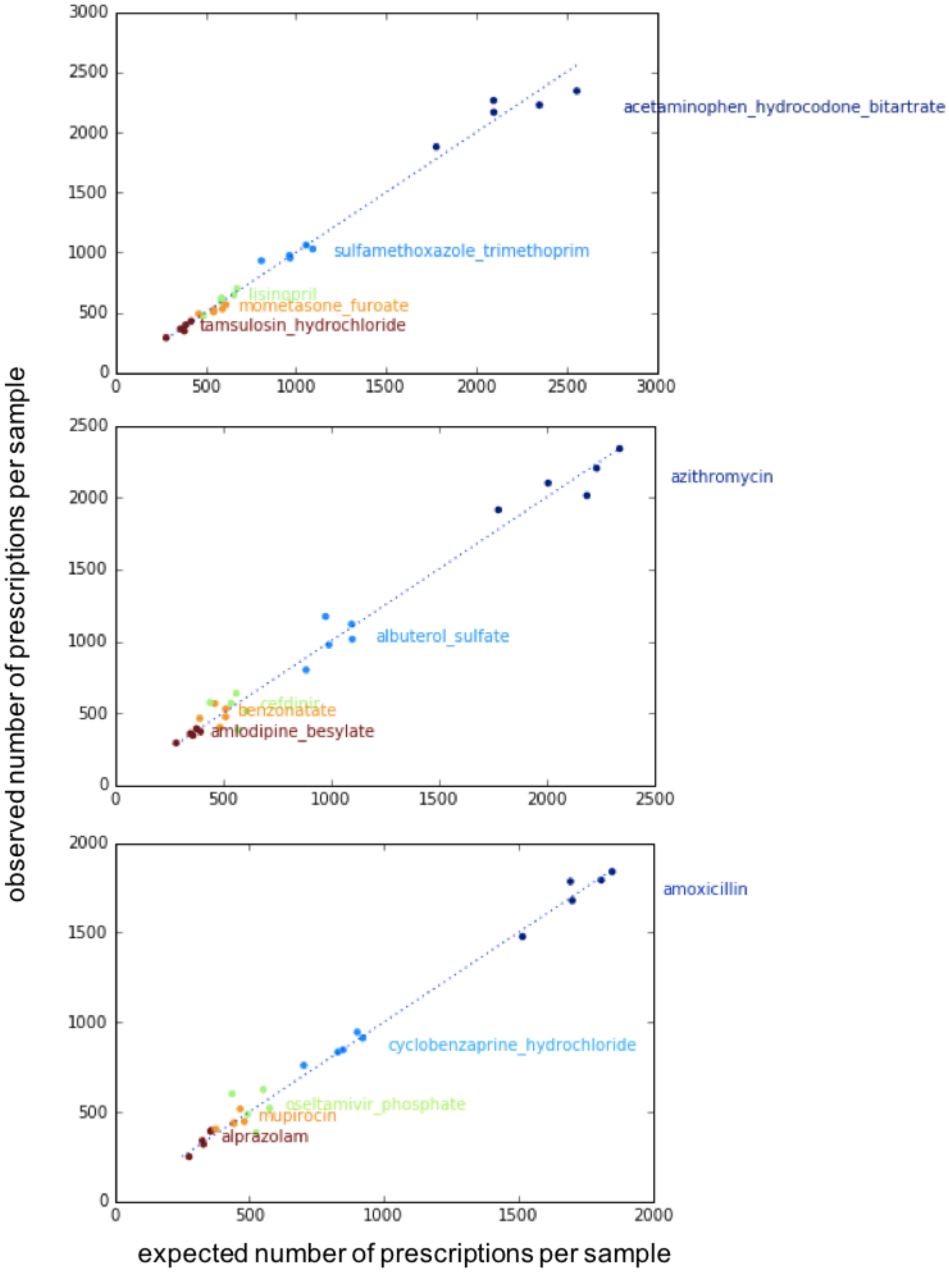

Supplementary Figure 11: We create 5 random samples of 50000 patients, who were held out from training of the model. We predict for each of the 5 samples, how many new prescriptions for each of the above drugs will be present. Each drug is indicated in a different color per subplot. Then, we compare the expected number (x-axis) to the observed number (y-axis), showing that our predictions are unbiased (dashed line). The visualization is only split into 3 subplots to avoid crowding.

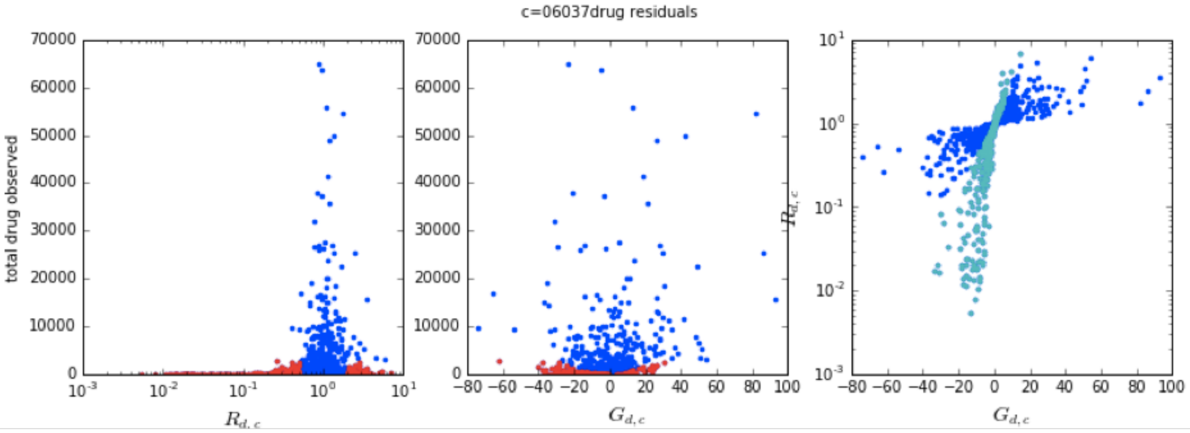

Supplementary Figure 12: Investigating the effect of drug rareness on residual scores. On the left, the ratio $R_{d,c} = y_{d,c} / \hat{y}_{d,c}$ (x-axis) for LA county is compared against total drug observed in that county, with red highlighting rare drugs. In the middle, the deviance value for each drug (described in methods), $G_{d,c}$, is shown. Finally, the right plot compares the two scores, higligting the rare drugs. The ratio has much more extreme values for the rare drugs.

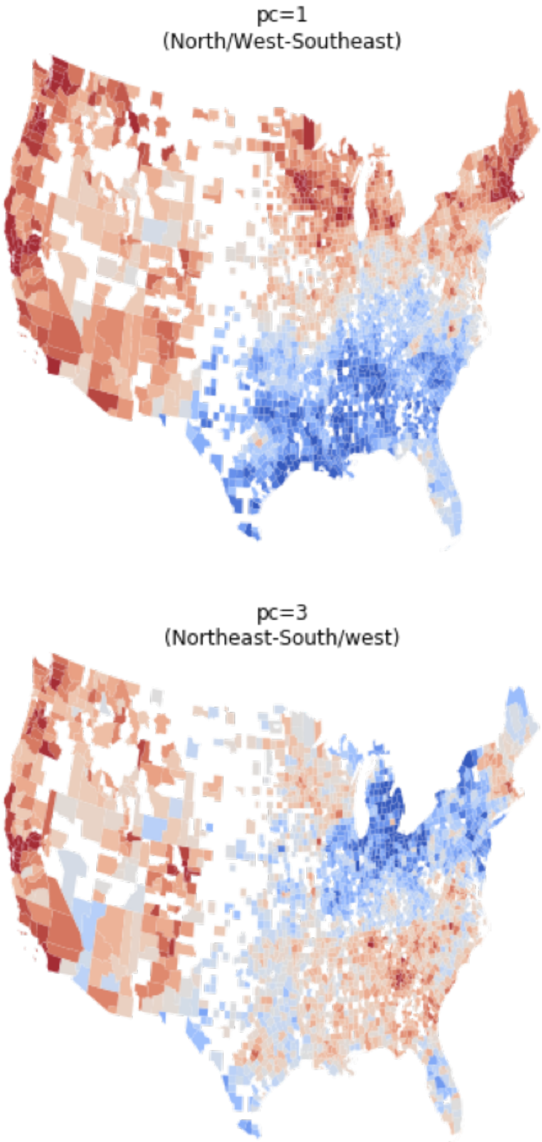

Supplementary Figure 13: The top 4 PCA components for analysis of males. We indicate in parentheses which component from the main text analysis each of these strongly correlates with.

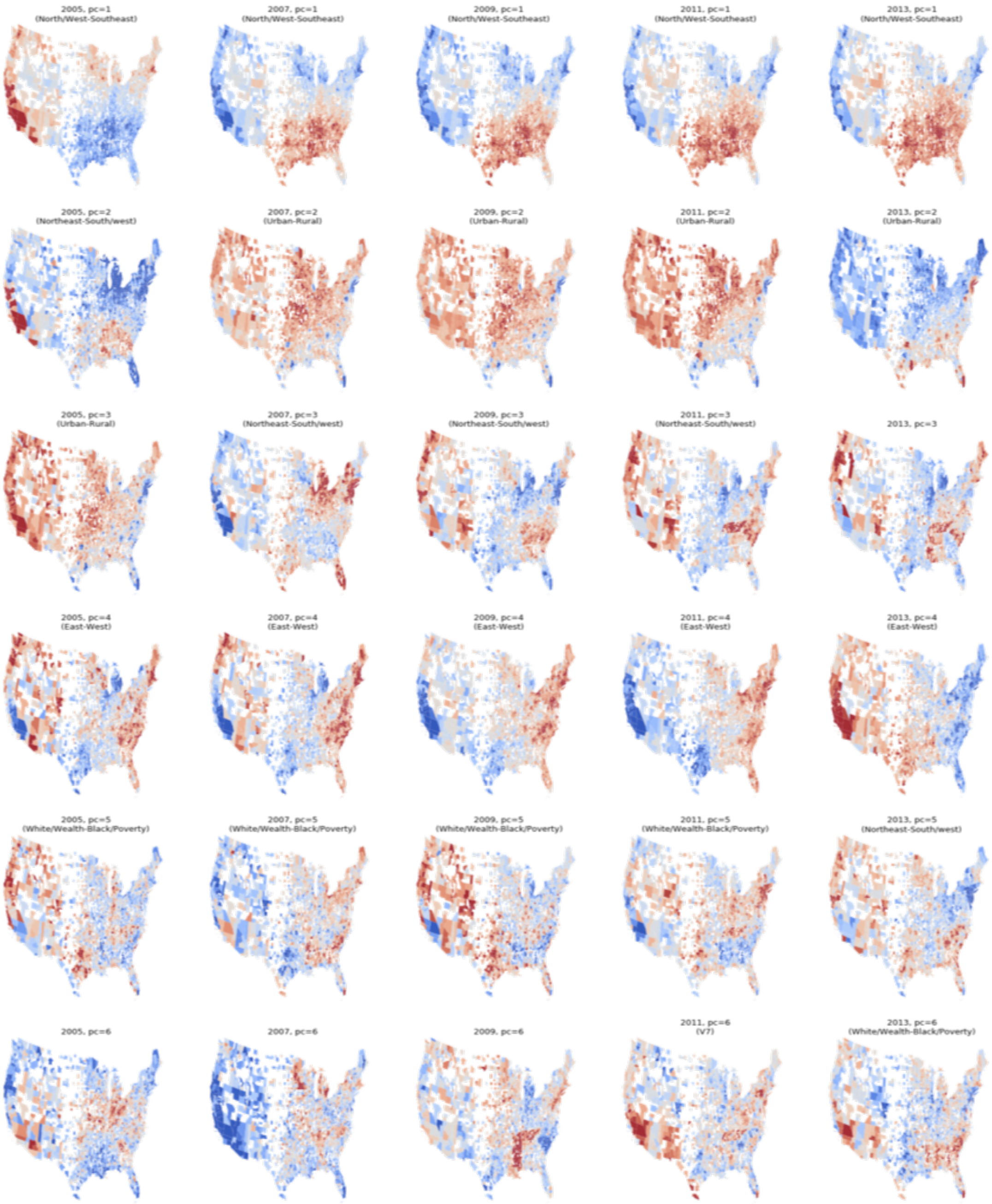

Supplementary Figure 14: Each column represents county projections using data from one year. We examine incident drug prescription in single-year periods, every two years. For each period (column), we use the prescriptions observed to calculate drug-county deviances, and the resulting principal component analysis. We show the top 6 components (rows). For components with strong correlation to a component from the full data analysis (using all years together), we indicate which full-data component, if any, the one-year component is correlated with.

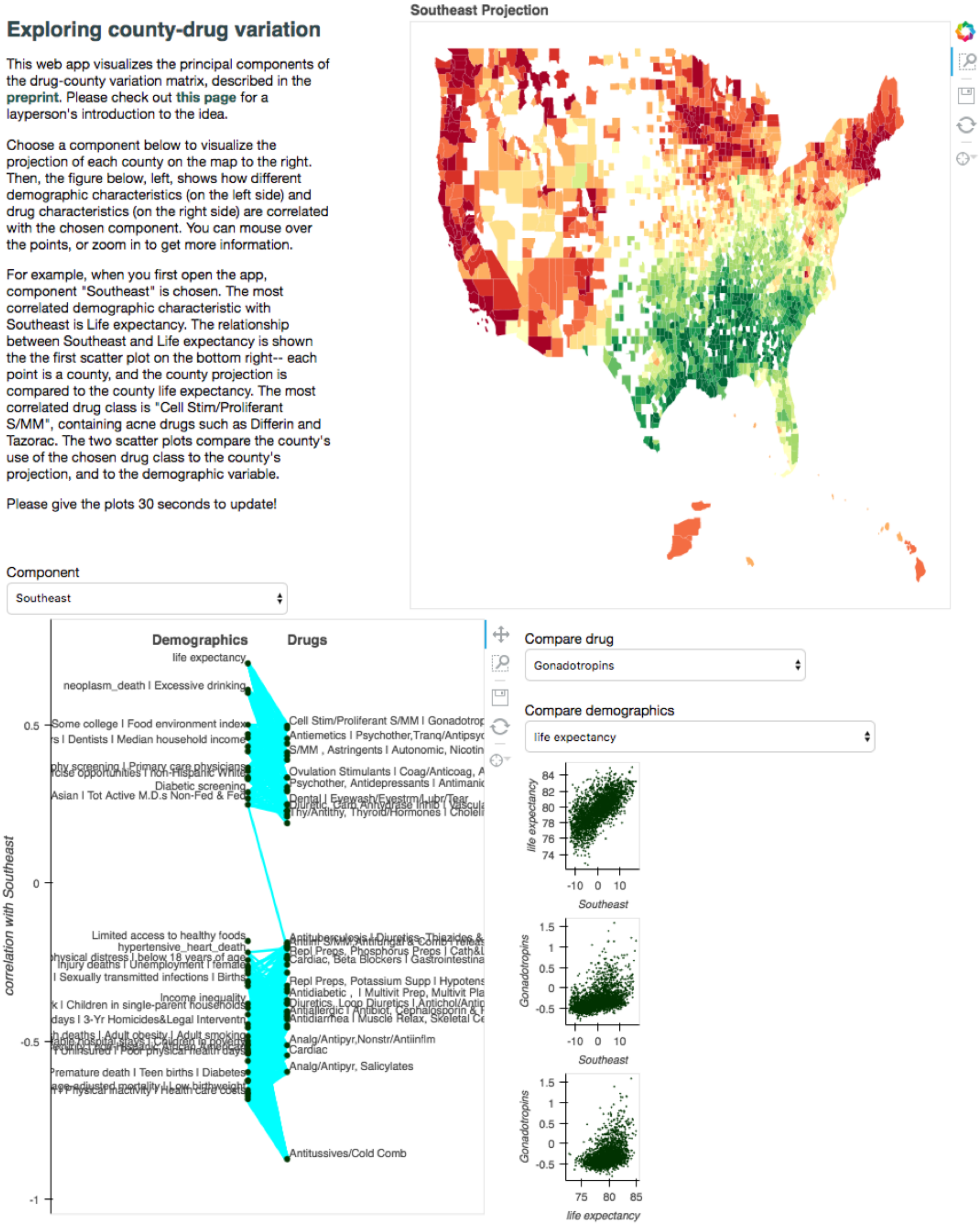

Supplementary Figure 15: Screenshot of the webapp at drugmap.uchicago.edu

Supplementary Table 1: For each cause of death, we perform regularized regression to identify drug deviances that are predictive of the cause of death. Then for causes of death with obviously related sets of drugs (curated below), we test whether these have significantly high regression coefficients using the rank-sum test (t-statistic and p-value shown).

cause of death	matched drugs	t-statistic	p-value
**diabetes**	['pioglitazone_hydrochloride', 'insulin_gl	3.46022403	0.00053973
**heart**	['fosinopril_sodium', 'nifedipine', 'atorvas	2.57955888	0.00989266
**hypertensive_heart**	['fosinopril_sodium', 'nifedipine', 'guanfa	3.06552706	0.00217287
**ischaemic_heart**	['atorvastatin_calcium', 'colesevelam_hy	2.92863387	0.00340455
**lower_respiratory**	['levalbuterol_tartrate', 'albuterol_sulfat	2.42508919	0.01530463

Supplementary Table 2: Results of canonical correlation analysis, from R package CCP, as described in the Method.

id	stat	approx	df1	df2	p.value
**1**	3.69E-06	7.60236333	5460	72773.5238	0
**2**	2.59E-05	6.4934578	5238	70232.6224	0
**3**	0.00011878	5.66688038	5018	67686.577	0
**4**	0.00044816	4.95008847	4800	65135.4455	0
**5**	0.00131051	4.38801156	4584	62579.2906	0
**6**	0.0032108	3.92597626	4370	60018.1802	0
**7**	0.00686827	3.53692138	4158	57452.1878	0
**8**	0.01330668	3.19622855	3948	54881.3925	0
**9**	0.02172528	2.9695161	3740	52305.8793	0
**10**	0.03397698	2.75675153	3534	49725.7394	0
**11**	0.04796734	2.61520892	3330	47141.0707	0
**12**	0.06691868	2.46697923	3128	44551.9774	0
**13**	0.09026361	2.33369556	2928	41958.5713	0
**14**	0.11922771	2.2040195	2730	39360.9711	0
**15**	0.1528362	2.08942941	2534	36759.3035	0
**16**	0.19343417	1.97135746	2340	34153.7029	0
**17**	0.24028321	1.85721317	2148	31544.3123	0
**18**	0.29738633	1.72552906	1958	28931.2831	0
**19**	0.3593277	1.60483349	1770	26314.7759	0
**20**	0.42244985	1.50494668	1584	23694.9606	0
**21**	0.48475072	1.42738089	1400	21072.0166	0
**22**	0.54819447	1.35905054	1218	18446.1337	7.77E-15
**23**	0.61188258	1.30075343	1038	15817.5118	7.46E-10
**24**	0.67492047	1.25477756	860	13186.3617	1.13E-06
**25**	0.74376737	1.18534105	684	10552.9046	0.00084329
**26**	0.81542487	1.09271086	510	7917.37169	0.07941285
**27**	0.8851176	0.98285149	338	5280	0.57696064
**28**	0.95091831	0.81140073	168	2641	0.9614583

Supplementary Table 3: Number of person-years and number of prescriptions

value	female	male
Person-years total	36756566	26708315
Antiinfect, Antibiotics EENT	13711660	9081018
Adrenals & Comb	12616131	8520510
Analg/Antipyr, Opiate Agonists	7324554	5115198
Antitussives/Cold Comb	5775915	3335374
Analg/Antipyr,Nonstr/Antiinflm	5757899	3726447
Antibiot, Penicillins	5050674	3736822
Misc Therapeutic Agents	4598160	1960402
Psychother, Antidepressants	3831749	1743679
Antiinf S/MM,Antifungal & Comb	3602509	1546034
Antibiot, Cephalosporin & Rel.	3515455	2465481
Gastrointestinal Drug Misc	2961973	1898259
Sympathomimetic Agents	2709015	1767817
Muscle Relax, Skeletal Central	2380988	1501532
ASH, Benzodiazepines	2150042	1074843
Antihyperlipidemic Drugs	2144466	2279010
Antiinf S/MM, Antiinf Loc Misc	1994801	840820
Contraceptive, Oral Comb	1914993	2443
Antiemetics	1878088	798208
Dental Agents	1815214	1072866
Sulfonamides & Comb	1714070	978577
Antivirals	1679571	982816
Anxiolytic/Sedative/Hypnot	1629590	919738
Analg/Antipyr, Salicylates	1601528	855793
Estrogens & Comb	1528070	4532
Diabetes Mell/Diab Supply	1343611	1085929
Anti-Infectives, Misc	1301776	448717
Urinary Anti-Infectives	1186213	77644
Cath&Lax, Laxatives/Enemas	1118166	891160
Cardiac, Beta Blockers	994735	915901
Anticonvulsants, Misc	993673	521451
Multivit Prep, Multivit Plain	975569	457526
Antiinfect, Antiinflam EENT	957570	628458
Cardiac, ACE Inhibitors	899564	976719
Antichol/Antimuscarin/Antispas	832918	362497
Pharmaceutical Aids/Adjuv	825158	634318
Eye/Ear/Nose/Throat Misc	766708	516229
Antidiabetic Agents, Misc	758486	729056

**Cardiac Drugs**	732303	660621
**Cardiac, Calcium Channel**	656731	621781
**Vitamin D**	633422	235448
**Antineoplastic Agents**	611184	274357
**Keratolytic Agents S/MM**	584593	408399
**Vaccines**	562278	395158
**Thy/Antithy, Thyroid/Hormones**	541185	154617
**Diuretics, Thiazides & Related**	519335	351842
**Psychother,Tranq/Antipsychotic**	505705	300332
**Stimulant, Amphetamine Type**	498083	480909
**Leukotriene Modifiers**	474528	323671
**Cell Stim/Proliferant S/MM**	472997	221331
**Histamine (H2) Antagonist**	472096	269641
**Vascular 5HT1 Agonist**	469824	107623
**CNS Agents, Misc.**	409182	250994
**Antiallergic Agents**	370764	236404
**Diuretics, Potassium-Sparing**	352011	137579
**Repl Preps, Potassium Supp**	344348	182906
**Diuretics, Loop Diuretics**	321193	213455
**Anticonvulsant, Benzodiazepine**	310221	161065
**Coag/Anticoag, Anticoagulants**	305505	293354
**Muscle Rel,Smooth-Genitour**	305142	106930
**Cardiac, Antiarrhythmic Agents**	292097	183449
**Antidiabetic Agents, Insulins**	284203	295315
**Immunosuppressants**	270182	112795
**Cath&Lax, Laxatives, Saline**	261735	183414
**Emoll/Moist/Demul/Protect S/MM**	214029	104074
**Anticholinergic**	211851	151190
**Antidiarrhea Agents**	210736	143508
**Hypotensive Agents**	203716	229101
**Antidiabetic Ag, Sulfonylureas**	200283	224809
**Autonomic, Nicotine Preps**	198248	173809
**Eyewash/Eyestrm/Lubr/Tear**	172339	58027
**Vasodilating Agents**	157670	219825
**Fluoride Preparations**	145444	111897
**Antiinf S/MM, Scabic/Pediculic**	143593	91867
**Antimalarial Agents**	137530	65229
**Repl Preps, Phosphorus Preps**	115066	66909
**Antiplatelet Agents**	104615	182009
**Antigout Agents**	85447	244687
**Sulfones**	72592	21835
**Gonadotropins**	69426	1460
**S/MM Misc, Astringents**	66143	33299

**Muscle Relax, Skeletal, Misc**	65395	41828
**Ammonia Detoxicants**	63535	40979
**Vitamin A & Derivatives**	44623	51223
**Ovulation Stimulants**	42502	4321
**Cardiac, Alpha-Beta Blockers**	41166	12355
**Oxytocics**	39685	321
**Anthelmintics**	38624	27526
**Diuretic, Carb Anhydrase Inhib**	36499	26491
**Antiinf S/MM,Antivirals & Comb**	36118	13279
**Keratoplastic Agents S/MM**	30337	30100
**Antituberculosis Agents**	28831	26525
**Antimanic Agents**	27821	17921
**Thy/Antithy, Antithyroid Agent**	25691	6956
**Cholelitholytic Agents**	24096	11148
**Cardiac, Cardiac Glycosides**	22208	31892
**Depig/Pig S/MM Depigment Agent**	21121	2164
**Parathyroid Hormones**	17907	2383
**Analy/Antipyr,OpiatePart Agnst**	15064	22096
**Digestants & Comb**	15063	11022
**Parasympathomimetic**	14290	12754
**Anticonv, Hydantoin Derivative**	12222	12164
**Pituitary Hormones**	12033	21546

Supplementary Table 4: Sources of demographic data

value	source
heart_death	http://wonder.cdc.gov/ucd-icd10.html
cerebrovascular_death	http://wonder.cdc.gov/ucd-icd10.html
flu_pneumonia_death	http://wonder.cdc.gov/ucd-icd10.html
diabetes_death	http://wonder.cdc.gov/ucd-icd10.html
suicide_death	http://wonder.cdc.gov/ucd-icd10.html
alzheimers_death	http://wonder.cdc.gov/ucd-icd10.html
neoplasm_death	http://wonder.cdc.gov/ucd-icd10.html
hypertensive_heart_death	http://wonder.cdc.gov/ucd-icd10.html
ischaemic_heart_death	http://wonder.cdc.gov/ucd-icd10.html
lower_respiratory_death	http://wonder.cdc.gov/ucd-icd10.html
Premature death Value	http://www.countyhealthrankings.org/rankings/data
Poor or fair health Value	http://www.countyhealthrankings.org/rankings/data
Poor physical health days Value	http://www.countyhealthrankings.org/rankings/data
Poor mental health days Value	http://www.countyhealthrankings.org/rankings/data
Low birthweight Value	http://www.countyhealthrankings.org/rankings/data
Adult smoking Value	http://www.countyhealthrankings.org/rankings/data
Adult obesity Value	http://www.countyhealthrankings.org/rankings/data
Food environment index Value	http://www.countyhealthrankings.org/rankings/data
Physical inactivity Value	http://www.countyhealthrankings.org/rankings/data
Access to exercise opportunities Value	http://www.countyhealthrankings.org/rankings/data
Excessive drinking Value	http://www.countyhealthrankings.org/rankings/data
Alcohol-impaired driving deaths Value	http://www.countyhealthrankings.org/rankings/data
Sexually transmitted infections Value	http://www.countyhealthrankings.org/rankings/data
Teen births Value	http://www.countyhealthrankings.org/rankings/data
Uninsured Value	http://www.countyhealthrankings.org/rankings/data
Primary care physicians Value	http://www.countyhealthrankings.org/rankings/data
Dentists Value	http://www.countyhealthrankings.org/rankings/data
Mental health providers Value	http://www.countyhealthrankings.org/rankings/data
Preventable hospital stays Value	http://www.countyhealthrankings.org/rankings/data
Diabetic screening Value	http://www.countyhealthrankings.org/rankings/data
Mammography screening Value	http://www.countyhealthrankings.org/rankings/data
Some college Value	http://www.countyhealthrankings.org/rankings/data
Unemployment Value	http://www.countyhealthrankings.org/rankings/data
Children in poverty Value	http://www.countyhealthrankings.org/rankings/data
Income inequality Value	http://www.countyhealthrankings.org/rankings/data
Children in single-parent households Val	http://www.countyhealthrankings.org/rankings/data
Social associations Value	http://www.countyhealthrankings.org/rankings/data
Injury deaths Value	http://www.countyhealthrankings.org/rankings/data
Drinking water violations Value	http://www.countyhealthrankings.org/rankings/data
Severe housing problems Value	http://www.countyhealthrankings.org/rankings/data
Driving alone to work Value	http://www.countyhealthrankings.org/rankings/data
Long commute - driving alone Value	http://www.countyhealthrankings.org/rankings/data
Premature age-adjusted mortality Value	http://www.countyhealthrankings.org/rankings/data
Frequent physical distress Value	http://www.countyhealthrankings.org/rankings/data
Frequent mental distress Value	http://www.countyhealthrankings.org/rankings/data
Diabetes Value	http://www.countyhealthrankings.org/rankings/data
Food insecurity Value	http://www.countyhealthrankings.org/rankings/data
Limited access to healthy foods Value	http://www.countyhealthrankings.org/rankings/data
Motor vehicle crash deaths Value	http://www.countyhealthrankings.org/rankings/data
Insufficient sleep Value	http://www.countyhealthrankings.org/rankings/data
Health care costs Value	http://www.countyhealthrankings.org/rankings/data
Median household income Value	http://www.countyhealthrankings.org/rankings/data
Residential segregation non-White/Whi	http://www.countyhealthrankings.org/rankings/data
Population estimate Value	http://www.countyhealthrankings.org/rankings/data
Percent of population below 18 years of	http://www.countyhealthrankings.org/rankings/data
Percent of population aged 65 years and	http://www.countyhealthrankings.org/rankings/data
Percent of population that is non-Hispan	http://www.countyhealthrankings.org/rankings/data
Percent of population that is American I	http://www.countyhealthrankings.org/rankings/data
Percent of population that is Asian	http://www.countyhealthrankings.org/rankings/data
Percent of population that is Native Haw	http://www.countyhealthrankings.org/rankings/data
Percent of population that is Hispanic	http://www.countyhealthrankings.org/rankings/data
Percent of population that is non-Hispan	http://www.countyhealthrankings.org/rankings/data
Population that is not proficient in Englis	http://www.countyhealthrankings.org/rankings/data
Percent of population that is female	http://www.countyhealthrankings.org/rankings/data
Tot Active M.D.s Non-Fed & Fed	http://www.countyhealthrankings.org/rankings/data
3-Yr Homicides&Legal Interventn	http://www.countyhealthrankings.org/rankings/data
Births	http://www.countyhealthrankings.org/rankings/data
life expectancy	http://www.healthdata.org/us-health/data-download
density	http://www2.census.gov/geo/docs/maps-data/data/gazetteer/Gaz_counties_national.zip
